# M2 macrophagy-derived exosomal miRNA-26a-5p induces osteogenic differentiation of bone mesenchymal stem cells

**DOI:** 10.1186/s13018-022-03029-0

**Published:** 2022-03-04

**Authors:** Zhang Bin-bin, Zha Xi Da-wa, Li Chao, Zhang Lan-tao, Wu Tao, Lu Chuan, Liu Chao-zheng, Li De-chun, Feng Chang, Wei Shu-qing, Dong Zu-nan, Pei Xian-wei, Zhi-xia Zhang, Li Ke-wen

**Affiliations:** 1 Department of Joint Surgery, Qinghai University Affiliated Hospital, Xining, 810000 Qinghai Province China; 2 State Key Laboratory of Military Stomatology & National Clinical Research Center for Oral Diseases & Shaanxi Key Laboratory of Stomatology, Department of Out-Patient, School of Stomatology, Fourth Military Medical University, Xi’an, 710032 China

**Keywords:** Exosomes, miRNA-26a-5p, Macrophages, Osteogenic differentiation

## Abstract

**Background:**

Bone marrow mesenchymal stem cells have always been a heated research topic in bone tissue regeneration and repair because of their self-renewal and multi-differentiation potential. A large number of studies have been focused on finding the inducing factors that will promote the osteogenic differentiation of bone marrow mesenchymal stem cells. Previous studies have shown that macrophage exosomes or miRNA-26a-5p can make it work, but the function of this kind of substance on cell osteogenic differentiation has not been public.

**Methods:**

M2 macrophages are obtained from IL-4 polarized bone marrow-derived macrophages. Exosomes were isolated from the supernatant of M2 macrophages and identified via transmission electron microscopy (TEM), western blotting, and DLS. Chondrogenic differentiation potential was detected by Alcian blue staining. Oil red O staining was used to detect the potential for lipogenic differentiation. And MTT would detect the proliferative capacity of cells. Western blot was performed to detect differential expression of osteogenic differentiation-related proteins.

**Results:**

The results showed that M2 macrophage exosomes will promote bone differentiation and at the same time inhibit lipid differentiation. In addition, M2 macrophage-derived exosomes have the function of promoting the expression of SOX and Aggrecan suppressing the level of MMP13. The exosome inhibitor GW4689 suppresses miRNA-26a-5p in M2 macrophage exosomes, and the treated exosomes do not play an important role in promoting bone differentiation. Moreover, miRNA-26a-5p can enable to promote bone differentiation and inhibit lipid differentiation. miRNA-26a-5p can promote the expression of ALP (alkaline phosphatase), RUNX-2 (Runt-related transcription factor 2), OPN(osteopontin), and Col-2(collagen type II). Therefore, it is speculated that exosomal miRNA-26a-5p is indispensable in osteogenic differentiation.

**Conclusions:**

The present study indicated that M2 macrophage exosomes carrying miRNA-26a-5p can induce osteogenic differentiation of bone marrow-derived stem cells to inhibit lipogenic differentiation, and miRNA-26a-5p will also promote the expression of osteogenic differentiation-related proteins ALP, RUNX-2, OPN, and Col-2.

## Introduction

Clinically, bone fracture is a common disease with a high degree of incidence, and a long healing cycle, which has brought a tremendously heavy financial burden to patients and families all over the world [1]. Considering that delayed or poorly fracture healing has still been a clinically unsolved problem, studying the mechanisms underlying fracture healing that will help to promote fracture healing is a necessity [2]. Bone mesenchymal stem cells (BMSCs) are crucial in the process of bone fracture healing under their osteogenic differentiation ability [3]. The differentiation of BMSCs is a combined effect of many factors, including non-coding RNA, immune cells, and cytokines. It has been reported that SNHG1, one of the longest non-coding RNAs, could attenuate osteogenic differentiation in BMSCs [4]. Similarly, miR-101 is a negative regulator of BMSCs [5]. In addition, macrophages are also necessary for osteoblast differentiation. Macrophages are one of the earliest cells to migrate into the fracture sites and play their role well in all stages of fracture healing [6, 7]. They can polarize into two distinctive subsets with unique functions: classically activated macrophages (M1) mainly functioned as pro-inflammation and alternatively activated macrophages (M2) mainly functioned as anti-inflammation [8]. Previous studies have shown that M2 macrophages could promote fracture healing, while M1 macrophages are more associated with a prolonged healing cycle [9, 10]. However, the mechanism by which the M2 type benefits the osteogenic differentiation remains unclear. Exosomes are 30–150 nm membrane vesicles secreted by most living cells and contain various proteins, genes (miRNA, mRNA), and lipids from original cells [11]. They have important biological functions and less cytotoxicity [12], so the potential application of exosomes in osteoblast differentiation/osteogenesis has attracted wide attention. Previous studies found that exosomes derived from a human periodontal ligament stem cell [13], endothelial progenitor cells [14], human CD34 + stem cells [15] can promote osteogenesis and accelerate bone regeneration. Furthermore, because of the importance of M2 macrophages in promoting fracture healing, we hypothesized that exosomes derived from M2 macrophages would be helpful to the osteogenic differentiation of BMSCs. Therefore, in this study, we put emphasis on the role of M2-derived exosomes in regulating the osteogenic differentiation of BMSCs.

## Methods

### Animals and macrophages generation

SD rats (10-week) were purchased from the Animal Center of Qinghai University. All animal experiments were approved by the Ethics Committee of Affiliated Hospital of Qinghai University. Bone marrow cells were isolated from the rats and then cultured in DMEM (Gibco, USA) with 10% FBS, 1% penicillin/streptomycin, and 50 ng/mL M-CSF for seven days. The isolated cells were routinely cultured in a 37 °C, saturated humidity, 5% CO2 incubator, and P4 generation cells were used for experiments. Therefore, bone marrow-derived macrophages (BMDMs) were harvested. In this experiment, 40 rats were used to obtain bone marrow-derived macrophages. The BMDMs were stimulated with IL-4 (20 ng/ml) for 24 h to differentiate from the M2 type. M2 macrophages were then harvested for further analysis.

### Flow cytometry

To identify M2 macrophages, BMDMs with or without IL-4 stimulation were harvested and stained with fluorochrome-conjugated antibodies against CD206 (GTX43682, genetex, China). The procedure was performed following the manufacturer’s instructions.

### Exosome purification and identification

After a 48 h fostering step, the fostering medium was collected and then centrifuged (300 g, 10 min, room temperature; then 12000 g, 40 min, 4℃). Subsequently, the supernatant was under ultracentrifugation twice at 100,000 g at 4 ℃ for 1 h and 10 min. The pellet containing exosomes were then subjected to density gradient centrifugation (100,000 g, 4 ℃, 16 h) for purification. Exosomes were observed to use transmission electron microscopy (Talos F200X S/TEM, Thermo ScientificTM). The NanoSight system was used to analyze the size distribution and concentration of exosomes. CD9, CD63, and CD81 can detect whether the exosomes were successfully extracted.

### Transfection assay

BMSCs (3 × 10^5^ cells/well) were seeded into 6-well plates,and until then the cells are completely attached and also replaced by a fresh medium for transfection. The miR-26a-5p mimic and NC mimic were synthesized from GenePharma (Shanghai, China). The miRNAs transfection was performed according to the instruction of lipofectamineTM2000 (Invitrogen, Carlsbad, CA). One microliter of the transfection reagent was diluted with 50 µL serum-free medium and incubated at room temperature for 5 min. Diluted miRNA mixed with diluted transfection reagent was incubated at room temperature for 20 min to form the miRNAs-liposome complexes. 100μL of miRNAs-liposome complexes was directed to each well of the cell culture plate, and the plate was then incubated at 37 °C, with 5% CO_2_ for 24–48 h.

### MTT assay

MTT kit (ab211091, abcam, UK) would measure cell viability according to the manufacture’s protocol. BMSCs were seeded to 96-well plates and then cultured for 48 h. Then, MTT kit was added into cells in medium for 4 h. After that, the absorbance (OD value) was measured at 570 nm.

### Detection of osteogenesis and chondrogenesis potential of BMSCs

BMSCs were cultured in a normal medium (NM) or osteogenesis induction medium (OM) containing 100 nM dexamethasone, 10 mM β-Glycerophosphate, and 50 mM ascorbate. Then alcian blue staining (ab150662, abcam, UK) and Oil Red O (aladdin, China) would be functioned as detector of the osteogenesis and chondrogenesis, respectively. Cells were fixed with 4% formalin, stained with Oil Red O staining solution for 15 min, or acidified with hydrochloric acid for 3 min, observed and collected pictures under a microscope. Besides, cells were stained with alcian blue staining solution for 30 min. After that cells were washed with distilled water for 15 min, and the cells were observed and analyzed under microscopy.

### ALP detection

Cells were lysed with a lysate without phosphatase inhibitors and then centrifuged to take the supernatant for the detection of ALP activity. The concentration of ALP in BMSCs was measured by the ALP activity detection kit (21101ES60, YEASEN, China) according to the manufacturer’s instructions.

### Western bolt

Total protein was extracted from cells of each group using RIPA lysis buffer (P0013, Beyotime), and BCA assay kit (P0009, Beyotime) measured the concentration of total protein. Prepared protein samples were separated by SDS-PAGE gels. Subsequently, proteins were transferred into 0.22 μm PVDF membranes and then blocked using 5% skimmed milk. The membranes were incubated with primary antibodies against CD9, CD63, CD81, SOX, Aggrecan, MMP-13, Col-2, Runx2, OPN, and ALP (Abcam, UK) overnight at 4℃. Next, the HRP-conjugated antibody was used as the secondary antibody. Finally, enhanced chemiluminescence (ECL) was used to visualize the membranes, with β-actin as an internal control.

### Real-time PCR

Total RNA from exosomes in M2 macrophages were extracted using Trizol reagent (cat. no. RR9109, TaKaRa). After quantification, the RNA was reversed to cDNA using PrimeScript™ RT reagent kit (cat. no. RR047A; Takara Bio, Inc.). Furthermore, mRNA expression was detected using TB Green TM Premix Ex TaqTM II (cat. no. RR820A; Takara Bio, Inc.) in the Applied Biosystems Prism 7300 Real-Time PCR System (Thermo Fisher Scientific, Inc.). The primers were as follows: miR-26a-5p-forward: GGTTCAAGTAATCCAGGATAGGCT, miR-26a-5p-reverse: CTCAACTGGTGTCGTGGAGTC. miR-U6-forward: CTCGCTTCGGCAGCACAT,

miR-U6-reverse: AACGCTTCACGAATTTGCGT.

### Statistical analysis

The data obtained in the experiment are all statistically analyzed by GraphPad Prism 8.0 software. T-test was selected between two groups and One-way ANOVA with Dunnett’s multiple comparisons among more than three groups expressed as $\overline{{\text{x}}}$ ± SD, each group was repeated 3 times independently, *p* < 0. 05 means the difference is significant.

## Results

### Characterization of exosomes derived from M2 macrophages

**Fig. 1:**
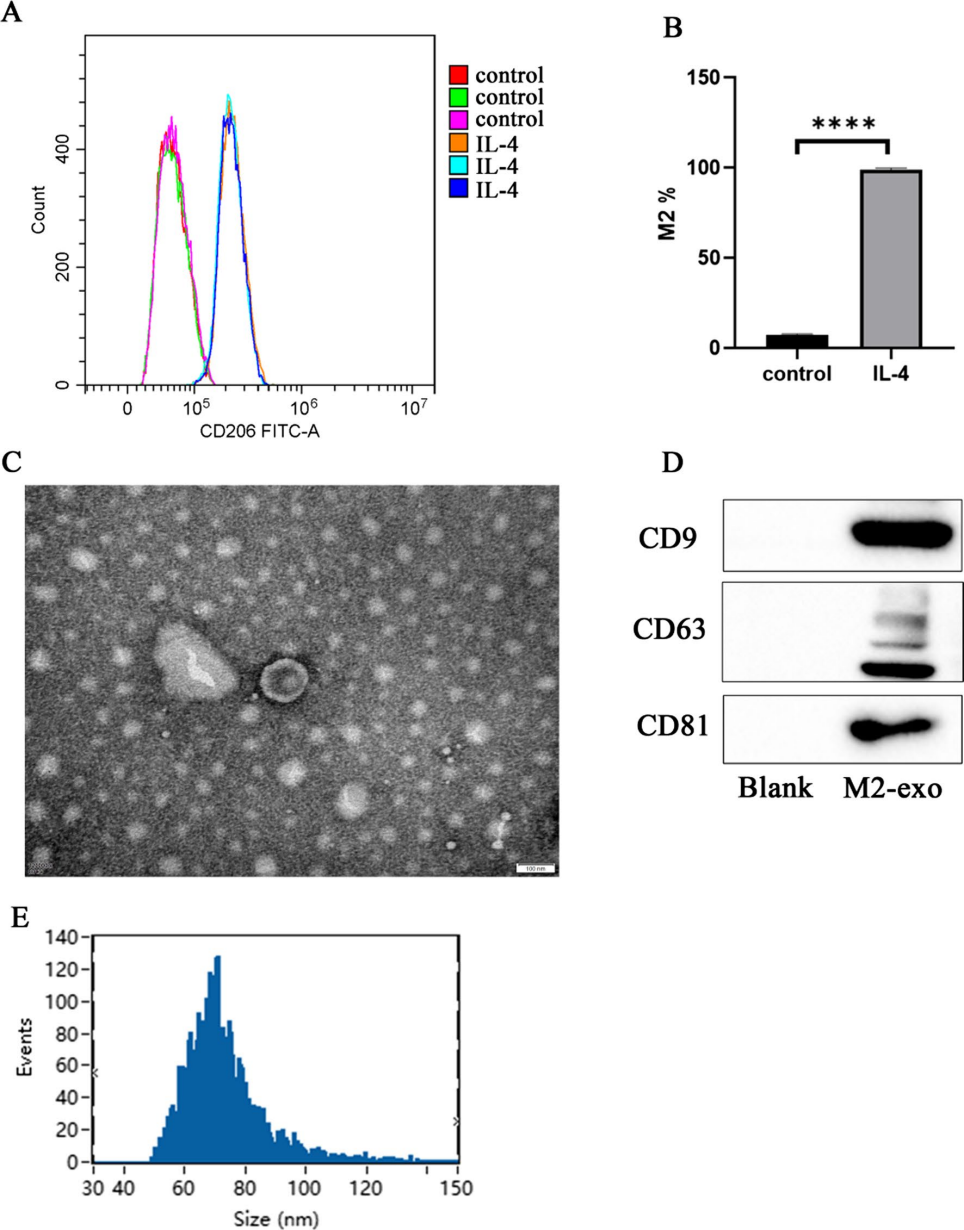
Identification of M2 type macrophages and exosomes. **A** Flow cytometric detection of CD206 expression in M0 and M2 type macrophages. **B** Flow cytometry statistics of M2 type macrophage differentiation. **C** TEM detection of the morphological structure of M2-exosomes promote (M2-exo). **D** Protein of exosomal marker. **E** Particle size distribution of M2-exo. *t* test was used for statistical analysis, **** means *p* ≤ 0.0001

To monitor macrophages polarization, we assessed the expression of phenotypic markers CD206 that is associated with M2 macrophages by flow cytometry. As shown in Fig. 1A, B, the rate of CD206 positivity in BMDMs was significantly increased after treatment with IL-4 compared with the control group. Next, the morphology and particle size of exosomes were analyzed. Exosomes were observed as closed circular vesicles by TEM (Fig. 1C). In addition, the markers CD9, CD63, and CD81 of exosomes were detected by western blot, which shows a strong positive expression in M2 exosomes (Fig. 1D). DLS was used to measure the distribution of M2 exosomes and revealed a peak of 73.38 nm (Fig. 1E). These results confirmed that the exosomes were successfully extracted from M2 macrophages supernatants.

### M2-exosomes promote the osteogenesis differentiation of BMSCs in hypoxic

**Fig. 2:**
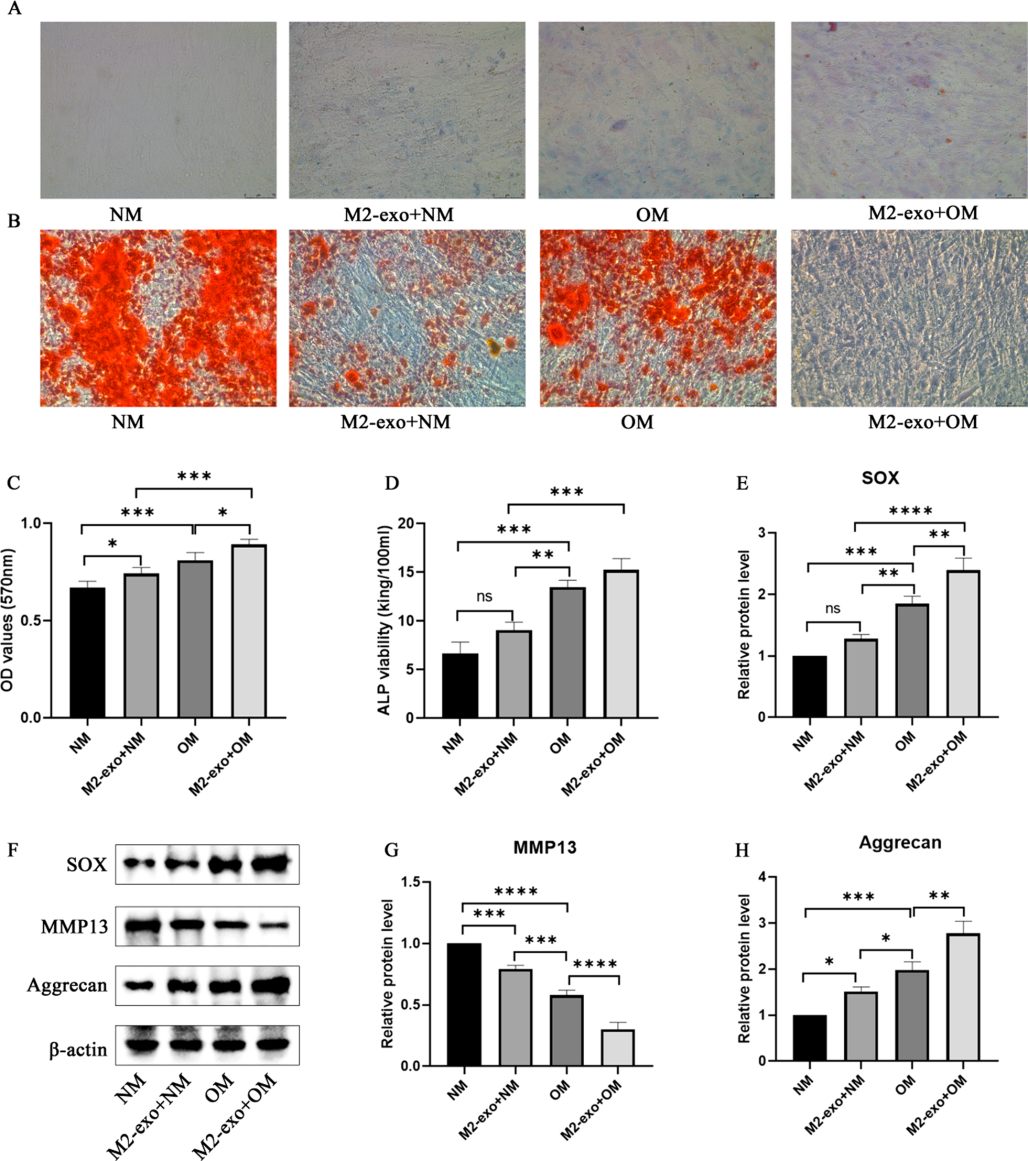
Osteogenic differentiation of BMSCs from bone marrow. **A** Alcian blue staining. **B** Oil Red staining. **C** BMSCs vitality. **D** ALP viability. **E**–**H** The proteins expression of SOX, MMP13, and Aggrecan. * means *p* ≤ 0.05, ** means *p* ≤ 0.01, *** and **** means *p* ≤ 0.0001

To prove the effect of M2-exo on the osteogenic differentiation of BMSCs, BMSCs were treated as M2-exo under hypoxic to test the effect on bone differentiation. As shown in Fig. 2A, compared with the NM group, BMSCs stained significantly deepen under the action of M2-exo and OM, respectively. Moreover, under the combined action of M2-exo and OM, the blue area is the most and the color is darkest. As shown in Fig. 2B, obvious red color was in the NM group, and lipid droplets were lightly stained and less dense under stimulation of M2-exo and OM, respectively. Moreover, under the combined action of M2-exo and OM, there was no obvious red. The results of cell viability experiments show that M2-exo and OM can induce cell proliferation (Fig. 2C). As for ALP, M2-exo and OM both have significantly been inducing effects on its activity (Fig. 2D). As shown in Fig. 2E–H, compared with the NM group, the expression levels of SOX and Aggrecan increased under the action of M2-exo and OM, respectively. Moreover, the expression level of M2-exo and OM was highest in the combined treatment group of M2-exo and OM. On the contrary, both M2-exo and OM have inhibitory effects on the expression of MMP13, and the expression level is the lowest in the combined treatment group of M2-exo and OM (Fig. 2E–H).

### miR-26a-5p promote the osteogenesis differentiation of BMSCs in hypoxic

**Fig. 3:**
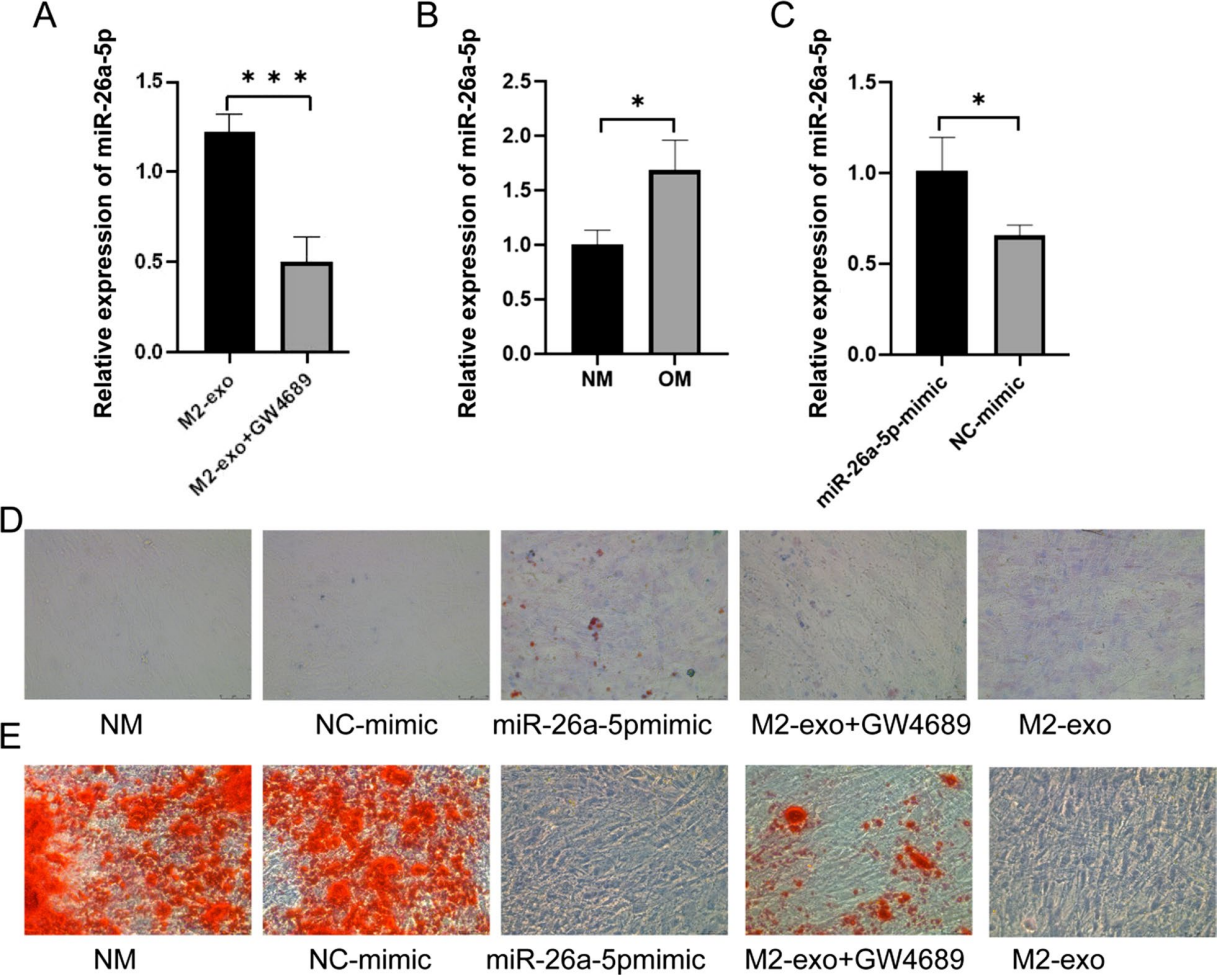
miR-26a-5p affects the osteogenic differentiation of BMSCs. **A** Relative expression of miR-26a-5p in M2-exo. **B** Relative expression of miR-26a-5p in BMSCs. **C** Relative expression of miR-26a-5p in BMSCs under treatment of miR-26a-5p mimic. **D** Alcian blue staining. **E** Oil Red staining. * means *p* ≤ 0.05, *** means *p* ≤ 0.0001

Macrophage exosomes can be swallowed by BMSCs, which carry a variety of miRNAs that may be the key to their functions. After GW4689 treatment, the content of miR-26a-5p derived from M2 macrophages decreased significantly (Fig. 3A). Compared with the NM group, under the induction of OM, the expression of miR-26a-5p in BMSCs cells increased significantly (Fig. 3B). Similarly, miR-26a-5p mimic can significantly induce the expression of miR-26a-5p (Fig. 3C).

To explore the effect of miR-26a-5p derived from M2 macrophages on the osteogenic and adipogenic differentiation of BMSCs, miR-26a-5p mimic and the supernatants of M2 macrophages treated with GW4689 were used to detect the effects on the osteogenic and adipogenic differentiation of BMSCs. As shown in Fig. 3D, compared with the NM and NC-mimic groups, the miR-26a-5p mimic transfection group had darker staining, but there was no obvious difference between NM and NC-mimic.In addition, compared with M2-exo, the staining of BMCs in M2-exo after treatment of GW4689 was reduced. However, comparing M2-exo and miR-26a-5p mimic groups, the degree of staining of BMSCs cells is similar. Oil red staining results showed that large areas of NC and NC-mimic cells were stained red. There were no red cells in the miR-26a-5p mimic transfection group and M2-exo incubation group, while the exosomes obtained from M2 macrophage after treatment of GW4689 incubated with BMSCs cells showing a small amount of red (Fig. 3E).

### The effect of miR-26a-5p on the potential pathway of osteogenic differentiation of BMSCs.

**Fig. 4:**
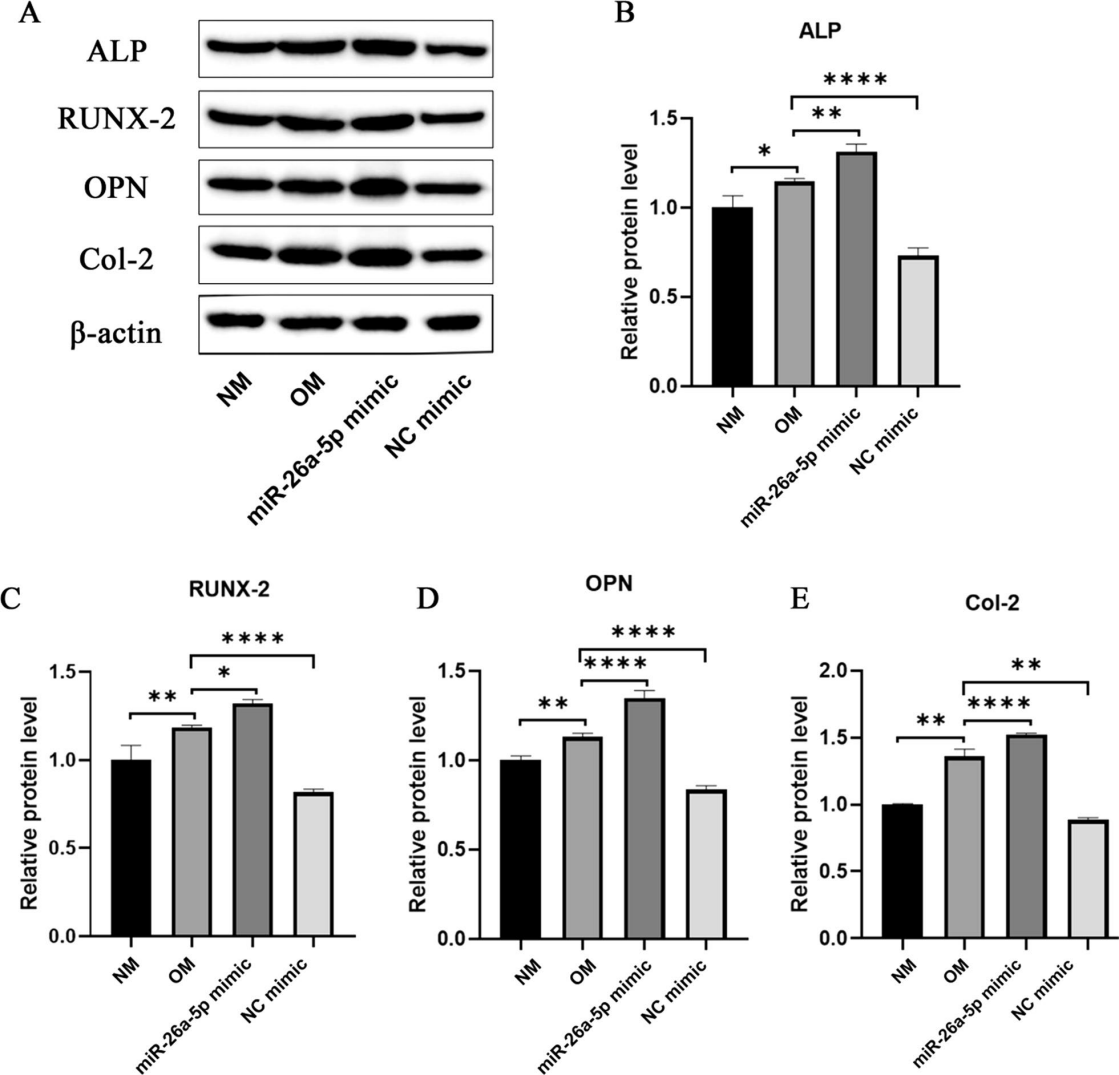
Effects of miR-26a-5p on BMSCs bone differentiation-related proteins. **A**–**E** Expression of bone differentiation related proteins ALP, RUNX-2, OPN, and Col-2. * means *p* ≤ 0.05, ** means *p* ≤ 0.01, *** and **** means *p* ≤ 0.0001

The proteins ALP, RUNX-2, OPN, and Col-2 all have a positive effect on osteogenic differentiation. As shown in Fig. 4, the expression levels of ALP, RUNX-2, OPN, and Col-2 in the NC mimic group and the NM group were similar. Compared with the NM group, OM and miR-26a-5p mimic both enable to promote the expression of these proteins, and the effect of miR-26a-5p mimic is better than OM.

## Discussion

Mesenchymal stem cells derived from bone marrow are the first mesenchymal stem cells which are more abundant than stem cells derived from other tissues [16]. BMSCs have the potential for self-renewal, multidirectional differentiation, differentiating from osteoblasts, adipocytes, chondrocytes, nerve cells, and other cells, which play an important role in osteogenic differentiation [17, 18]. Exosomes gained from M2 macrophages have multiple activities. For example, Yuan Xiong reported that exosomes isolated from the M2 macrophages are capable of accel arising fracture healing via BMSC differentiation in vitro and in vivo [10]. To this end, M2 macrophage exosomes and miR-26a-5p were used to demonstrate the effect on bone differentiation. Moreover, the influence of M2 macrophages on bone differentiation may be related to the secretion of miR-26a-5p carried by exosomes.

The positive role of M2 macrophages during bone fracture healing is already well known [10, 19]. Previous results suggested that M2D-Exos were able to stimulate osteoblast differentiation and bone mineral deposition, as well as to promote fracture healing in murine models [20]. This study successfully polarized rat-derived macrophages into M2 type in vitro, and successfully isolated and identified exosomes from M2-type macrophages. The expression of the marker proteins CD9, CD81, and CD63 were identified, and the particle size was determined to be about 73.38 nm.

Exosomes are a type of extracellular vesicles with a diameter between 30 and 150 nm [21]. Exosomes are formed in endosomal compartments with a large number of protein molecules, DNA, RNA, mRNA, microRNA, etc., which can pass through the intercellular delivery play an important role [22]. In this project, M2-exo induced osteogenic differentiation of BMSCs, inhibited the ability of adipogenic differentiation, promoted the expression of SOX and Aggrecan, and inhibited the level of MMP13. SOX9 and aggregate sugar have a repairing effect on the damaged proteasome function of human osteoarthritis chondrocytes [23, 24]. Extracellular matrix (ECM) remodeling is necessary for tissue development and self-healing, and its process is tissue-specific [25]. Matrix metalloproteinases (MMPs) play an important role in the remodeling of ECM. Matrix metalloproteinase 13 (MMP 13) is highly expressed during the osteogenic differentiation of human bone marrow mesenchymal stem cells [26]. In addition, the knockout of MMP 13 reduced the osteogenic differentiation of hMSCs grown on the Col-I matrix [27]. These results indicate that exosomes derived from M2 macrophages can promote bone differentiation.

In recent years, the function and potential application of miRNA in osteogenic differentiation have attracted more and more attention, and inhibition of exogenous microRNAs can regulate osteogenic differentiation [28]. Macrophages are also necessary for osteoblast differentiation in vitro and are important in both the early and late stages of fracture healing [19]. The macrophage phenotype must switch the more inflammatory M1 subtype to the anti-inflammatory M2 subtype during the healing process [9, 29]. Non-union is usually associated with a prolonged pro-inflammatory macrophage response [9, 29]. M2 macrophages perform functions that are usually related to their secretion of exosomes and miRNA carried by exosomes [30, 31]. In this study, after using the inhibitor GW4689, the miR-26a-5p in exosomes decreased. Both miR-26a-5p and M2-exo can promote bone differentiation and inhibit ester differentiation. After GW4689 processed macrophages, this positive effect of M2-exo disappeared. MicroRNA-26a-5p has a positive effect on the Wnt/Ca2 + pathway and osteogenic differentiation of mouse adipose-derived mesenchymal stem cells [32]. MicroRNA-26a-5p targets Wnt5a to regulate osteogenic differentiation of human periodontal ligament stem cells from the inflammatory microenvironment [33]. These results indicate that miR-26a-5p carried by exosomes from M2 macrophages can promote bone differentiation.

MicroRNA-26a-5p actively promotes bone differentiation related proteins ALP, RUNX-2, OPN, and Col-2. Osteoblasts formed by differentiation of BMSCs secrete a glycoprotein-alkaline phosphatase (ALP), which hydrolyzes phosphate esters during the process of bone formation and provides the necessary phosphoric acid for the deposition of hydroxyapatite [34]. The function of Runx2 has a vital relationship with the uptake of glucose. Glucose is the main nutrient of osteoblasts, which is mainly ingested through Glut1, and plays a key role in the growth and development of osteoblasts [35, 36]. OPN and Col-2 are osteogenic markers. OPN is abundant in bones and participates in the fracture healing process [37]. There is an area that is rich in aspartic acid in the OPN molecule, through which OPN can combine with the light apatite in the tissue to form bone minerals [38]. Additionally, the COL2A1 gene can encode type II collagen precursor(Col-2), which is a major protein that constitutes articular cartilage and hyaline cartilage [39]. Herein, we found that MicroRNA-26a-5p and osteogenesis induction medium has the same effect on ALP, RUNX-2, OPN, and Col-2. Therefore, miR-26a-5p may induce bone differentiation. However, the biological functions of miRNAs are inextricably linked to their downstream target genes. MiRNAs interact with the 3’untranslated region (3’UTR) of target mRNAs to induce mRNA degradation and translational repression. Thus, the failure to find the target gene for miRNA-26a-5p during osteogenic differentiation remains a problem in this experiment.

## Conclusion

In this study, miRNA-26a-5p in exosomes that were obtained from M2 macrophage can promote osteogenic differentiation and inhibit adipogenic differentiation. miRNA-26a-5p can promote the expression of osteogenic differentiation-related proteins ALP, RUNX-2, OPN, and Col-2. However, the differentiation and maturation of BMSCs are a complicated process. Whether other miRNAs or other non-coding RNAs participate in the process of M2 macrophage exosomes regulating the osteogenic differentiation of BMSCs remain to be further analyzed.

## Data Availability

The datasets used or analyzed during the current study are available from the corresponding author on reasonable request.
